# Polypharmacy Exposure, Aging Populations, and COVID-19: Considerations for Healthcare Providers and Public Health Practitioners in Africa

**DOI:** 10.3390/ijerph181910263

**Published:** 2021-09-29

**Authors:** Jamaji C. Nwanaji-Enwerem, Edward W. Boyer, Ayobami Olufadeji

**Affiliations:** 1 Gangarosa Department of Environmental Health, Emory Rollins School of Public Health, Atlanta, GA 30322, USA; 2 Department of Emergency Medicine, Emory University School of Medicine, Atlanta, GA 30322, USA; 3 Department of Emergency Medicine, Division of Medical Toxicology, Brigham and Women’s Hospital, Harvard Medical School, Boston, MA 02115, USA; eboyer@bwh.harvard.edu; 4 Beth Israel Deaconess Medical Center, Department of Emergency Medicine, Harvard Medical School, Boston, MA 02215, USA; aolufade@bidmc.harvard.edu

**Keywords:** pandemic, geriatrics, African, medicine, policy, shared decision making, registry, surveillance, pharmaceuticals

## Abstract

Given the continent’s growing aging population and expanding prevalence of multimorbidity, polypharmacy is an increasingly dire threat to the health of persons living in Africa. The COVID-19 pandemic has only exacerbated these issues. Widespread misinformation, lack of vaccine access, and attempts to avoid being infected have resulted in increases in Africans’ willingness to take multiple prescription and nonprescription medications and supplements. Issues with counterfeit pharmaceuticals and the relatively new recognition of emergency medicine as a specialty across the continent also create unique challenges for addressing this urgent public health need. Experts have called for more robust pharmaceutical regulation and healthcare/public health infrastructure investments across the continent. However, these changes take time, and more near-term strategies are needed to mitigate current health needs. In this commentary, we present a nonexhaustive set of immediately implementable recommendations that can serve as local strategies to address current polypharmacy-related health needs of Africans. Importantly, our recommendations take into consideration that not all healthcare providers are emergency medicine trained and that local trends related to polypharmacy will change over time and require ever-evolving public health initiatives. Still, by bolstering training to safeguard against provider availability biases, practicing evidence-based prescribing and shared decision making, and tracking and sharing local trends related to polypharmacy, African healthcare providers and public health practitioners can better position themselves to meet population needs. Furthermore, although these recommendations are tailored to Africans, they may also prove useful to providers and practitioners in other regions facing similar challenges.

## 1. COVID-19 and an Older Nigerian Couple

Lopinavir/Ritonavir: one tablet daily for two weeks, Azithromycin: 500 mg daily for two days, Ivermectin: five tablets daily for three days, Omeprazole: 20 mg daily for one week, Zinc: 100 mg daily for two weeks.

This medical regimen was prescribed to a 64-year old Nigerian woman—with a past medical history of hypertension—who was recently diagnosed with COVID-19 after presenting to her local clinic in Lagos with a headache, fatigue, and body aches. In her opinion, the number of medications prescribed was well beyond her regular lisinopril regimen; however, it seemed reasonable for fighting a disease as severe as COVID-19. She was willing to take any steps that would guarantee a speedy recovery. In the same household, her nearly 70-year old husband, who has no comorbidities, was coming to terms with his wife’s recent diagnosis. Although he was doing a reasonable job at self-isolating, he also turned to WhatsApp for strategies that would mitigate his risk of infection. In addition to daily hot water mist treatments, he had begun a regimen of eight supplements including salvia extract tablets. Similar to his wife, he too was willing to take any steps that would guarantee that he would not be infected.

Although this is the story of one older couple living in Lagos, Nigeria, their sentiments are likely shared with many people across the globe during the COVID-19 pandemic. With a lack of therapeutics, limited vaccine access, rising infection, hospitalization, and death rates, individuals are willing to go to many lengths to avoid infection. In many minds, taking some form of medication or supplement—even multiple medications—offers considerable benefit. However, this calculation has not only proven itself to be incorrect, in some instances it can prove itself to be deadly [1]. 

## 2. Polypharmacy in the African Context

Polypharmacy is often defined as the regular use of multiple (usually ≥ 5) medications by a single individual, and remains an important public health matter because it carries the risk of adverse drug reactions [2]. Although polypharmacy is primarily viewed as a concern in older populations and younger at-risk individuals—both of which are more likely to suffer multimorbidity—it has become a more urgent concern for all populations during the COVID-19 pandemic [3]. According to the World Health Organization’s Africa Office, just over 1% of Africans are fully vaccinated against COVID-19. Moreover, experts predict that it may take until 2023 for the vaccine to reach some of the world’s poorest countries [4]. Similar to the woman in the aforementioned vignette, given limited vaccine access, individuals living in some parts of the world who are already taking medications for chronic diseases now find themselves being prescribed antibiotic, antiviral, and antiparasitic agents to help manage their COVID-19 infections. Furthermore, similar to the husband in the vignette, individuals without preexisting conditions are subjecting themselves to a multitude of nonprescription agents. Both prescription and nonprescription polypharmacy carry very important risks. First, antibiotics, antivirals, and antiparasitic medications presently do not have a widely supported therapeutic role in COVID-19 management [5]. Hence, their benefits to COVID-19 patients are minimal at best. On the contrary, the risks of the medications—although they are seemingly ignored—remain consequential. For instance, adverse neurological events associated with the use of ivermectin, a drug primarily used as an antiparasitic agent, remain a concern [6]. Ivermectin is metabolized by Cytochrome P450 (CYP450) enzymes in the liver. As a result, prescribing ivermectin along with other CYP450 inhibitors such as statins (for cholesterol) or lopinavir / ritonavir (HIV protease inhibitors that are also being mis-prescribed for COVID-19) contributes to the potential individual and shared toxicity of these agents [7]. Issues with nonprescription supplements can be more unsettling because of the limited regulation already surrounding these compounds. Individuals may feel that these compounds are safer because they do not require physician prescriptions; nevertheless, many supplements may have active agents with profound biological effects and toxicity. In the United States, an estimated 23,000 emergency department visits each year can be attributed to adverse events related to dietary supplements [8]. 

Although the polypharmacy-related concerns of drug–drug interactions and deregulated nonprescription supplements are relevant globally, there are specific considerations that make polypharmacy in Africa an even greater urgent and emerging public health concern. Older age is one of the primary risk factors for polypharmacy, given that older individuals are more likely to suffer from multimorbidity. Africa’s older population is expected to triple from 74.4 million in 2020 to 235.1 million in 2050—growing faster than any other region across the globe [9]. As such, diseases and associated conditions of older populations, including polypharmacy, must become a larger priority for healthcare providers and public health practitioners on the continent. Additionally, although we described the many unknowns surrounding nonprescription supplements, Africa has the highest prevalence of falsified and substandard prescription pharmaceuticals [10]. Reports demonstrate the presence of counterfeit medications for numerous conditions, including diabetes, HIV/AIDS, bacterial infections, and pain [10]. Counterfeits for COVID-related drugs, including chloroquine and vaccines, are only among the most recent to follow this existing trend [11]. Thus, the risks of adverse drug reactions due to polypharmacy of substandard drugs in Africa may be among the highest in the world. Finally, emergency medicine as a standalone specialty is still in its infancy throughout most of Africa [12]. Consequently, the widespread ability of healthcare providers who are trained to specially treat toxicological presentations and acute drug–drug interactions is limited. 

## 3. Strategies for African Providers and Public Health Practitioners

Just as COVID-19 has highlighted the urgent need to address multiple longstanding public health issues, it has also placed a spotlight on the management of polypharmacy in Africa [13,14]. Moreover, given Africa’s growing aging population and unique issues with counterfeit medications and emergency care services, this matter is likely to be of even greater consequence in the future. Wider government information campaigns, improved regulation of pharmaceuticals, and continued improvements in healthcare infrastructure, including more investments in emergency medicine residency programs, are among the long-term efforts necessary to help meet present needs. At the same time, people on the African continent currently suffer from the threat of polypharmacy. The following are our nonexhaustive recommendations that can be employed immediately at the local level to help alleviate some of this burden.

### 3.1. Bolstering Training to Safeguard against Provider Availability Biases

Providers should include polypharmacy in their differential diagnoses. Emergency care providers often fall victim to availability bias. As of 5 August 2021, over 6.8 million COVID-19 cases had been identified in Africa and there were over 170,000 reported deaths. Hence, it is reasonable to deduce that patients presenting with fatigue, abdominal pain, and nausea could be presenting with a mild form of COVID-19. In resource-limited environments where testing is not available [15,16], the likelihood of this diagnosis being inappropriately determined is increased. Such has been the case in instances of malaria overdiagnosis in Africa [17]. Deficits in diagnostic testing and assumed high prevalence of disease may have resulted in missed opportunities to accurately diagnose and treat other etiologies of fever [18,19]. Hence, this same presentation of fatigue, abdominal pain, and nausea could be COVID-19-related or it could be due to another etiology such as polypharmacy—especially in a patient who has recently started a series of agents to prevent COVID-19 infection—and could result in more serious sequalae if not properly addressed. Subacute acetaminophen/paracetamol poisoning can present with the common viral symptoms of nausea, pallor, and sweating. However, acetaminophen/paracetamol poisoning can result in frank liver failure if not promptly diagnosed and treated. Performing a thorough medical history, including a review of prescription and nonprescription agents, will be crucial in making this important distinction. For a patient’s primary care provider, this is an opportunity to deprescribe any medications that are no longer necessary or lack utility. 

The leadership of a medical group or practice, especially those that are not emergency medical care, should consider implementing brief or refresher toxicology curricula. For providers working in clinics near instituitions with emergency departments, brokering local partnerships may prove to be a fruitful endeavor. For providers without access to local licensed medical toxicologists, online resources can be useful. For instance, as part of the Global Education Toxicology Uniting Project (GETUP), the American College of Medical Toxicology provides online access to introductory toxicology course modules. The resources require computer and internet access, but are offered at no additional cost (https://www.acmt.net/Intro_Toxicology_Course.html; accessed on 6 August 2021). For more junior staff and trainees, a free medical student emergency medicine course offered by Lecturio and the International Emergency Medicine Education Project may be useful (https://iem-course.org/courses/emergency-medicine-cc/ accessed on 6 August 2021). Notwithstanding, there remain important issues with internet and electricity penetrance throughout the continent [20]. Thus, through regular communication between more technologically equipped providers/practitioners and their rural and local community health worker counterparts, the knowledge from these online resources will likely need to be disseminated via efforts such as mobile clinics [21,22] and “health centers by phone” SMS-based programs [23] that have proved useful for improving health access in rural areas.

### 3.2. Continue the Practice of Evidence-Based Prescribing and Shared Decision Making

Providers must continue to support the dogma that randomized controlled trials (RCTs) should guide clinical practice—not observational human studies, case reports, or experimental animal/cell studies. It is simply a matter of patient safety, especially for those in a profession devoted to “first, do no harm.” The pressure that providers feel to “give patients something” is understandable when patients present with viral symptoms, particularly during the COVID-19 pandemic. Nevertheless, prescribing an antibiotic for a viral etiology offers little benefit and may carry significant risks [24]. Such practices contribute to antibiotic resistance in the community as well as the culture of polypharmacy [25]. One strategy to relieve prescribing pressures involves embracing shared decision making (SDM) in patient care and sharing your clinical reasoning with patients. Prior studies of SDM in African health settings have identified a lack of decision aids/tools as an important barrier to effective SDM [26]. Where possible, providers should try to supply their patients with culturally and education-level-appropriate materials during SDM conversations. These materials (e.g., handouts and pamphlets) can facilitate on-the-spot patient education and serve as a “tangible” product of the patient encounter. Additionally, reputable information can help dilute misinformation found in the community. Clinics can use premade resources for diseases from organizations such as the United Nations; however, population-tailored aids may be more acceptable to recipients. Africa is not monolithic and even within one country a wide range of cultural and religious beliefs exists. SDM frameworks help to create an environment where patients can share their cultural beliefs. Providers should be cognizant of being respectful towards patient’s beliefs, even when scientifically unfounded. Nonetheless, providers should also be empowered to respectfully and directly tell patients when practices are not supported by the field and could have negative impacts on the patient’s health. SDM frameworks also provide opportunities for including family members in decision making, subsequently amplifying the reach of patient education efforts. This is particularly important for older patients whose family members often serve as caregivers. Institutions such as the Ottawa Hospital Research Institute provide free resources for patient decision aids (https://decisionaid.ohri.ca; accessed on 6 August 2021), and work by authors such as Jull et al. (2015) provide insights on how to adapt SDM tools for non-Western cultures [27]. 

Returning to the use of ivermectin, it is important to note that on 11 February 2021, the United States National Institutes of Health (NIH) COVID-19 Treatment Guidelines Panel updated their stance on ivermectin to: “there are insufficient data to recommend either for or against the use of ivermectin for the treatment of COVID-19 [28].” Although some are interpreting this change to mean that ivermectin can be used to treat COVID-19, in line with the recommendation, we believe that there is not enough evidence to make that assertion. This may change, but at present there is not a solid evidenced-backed consensus that ivermectin should be used to treat COVID-19. In situations where patients are very sick, and physicians are electing to use ivermectin off-label, in the spirit of SDM, they should be sure to convey these unknowns to their patients. Again, it is a matter of patient safety that patients are informed of the associated risks of their respective medications. Notably, at this moment, the World Health Organization (WHO) advises that ivermectin only be used to treat COVID-19 (of any severity) in clinical trials—not everyday practice. Presently, WHO also has a strong recommendation against the use of hydroxychloroquine and lopinavir/ritonavir for treatment of COVID-19 of any severity [29]. Providers should also make an effort to remain up-to-date on COVID-19 treatment guidelines [5].

### 3.3. Formally Track and Share Local Trends and Patterns

No one is more knowledgeable about a healthcare ecosystem than the people who live in it and interact with it every day. Just as word travels about the best restaurants, tailors, or mechanics, people also discuss healthcare institutions including clinics and pharmacies. For example, relatives and friends can have striking influence on a person’s selection of a healthcare provider. Similar interactions can be formally leveraged as a means of improving overall quality of care. We encourage providers who have identified a patient experiencing an adverse drug reaction to determine where the patient received his / her medications or supplements. By maintaining records of this information for periodic review, clinicians can help identify trends that can be shared with patients, or among clinics in a city or town. Public health practitioners skilled in population health and epidemiology can be an invaluable resource for studying trends and communicating them to the government and broader public. These efforts may also contribute to the development of formalized surveillance registries, which are believed to be critical for improving the quality of healthcare in low- and middle-income countries [30]. Although clinicians and researchers often have limited authority to regulate problematic pharmacies, physicians can refer or recommend that patients go to more trusted sources. Public health practitioners can work to establish bulletins to inform the public of concerning findings. Making such practices the norm also creates an additional layer of accountability for African health systems. Pharmaceutical merchants who believe that their reputation depends on the quality of their merchandise may be more considerate about their suppliers and less inclined to sell fraudulent medications. Research performed in Ghana has documented the dual role that social capital/relationships can play in encouraging or discouraging patients from embracing behaviors [31]. The tracking and sharing of reputable pharmacies provides an additional opportunity to counteract social media misinformation and other health adverse forms of social interaction with something health positive. Reports of a feared loss of power and prestige as impediments to referrals in African health systems also exists as providers see little incentives in referring patients elsewhere [32]. While this is less of a concern for emergency services given the nature of the specialty, it may be a continued concern for physicians of other specialties simply providing emergency care. Being viewed as a reputable source of information/referrals—especially with pharmacies—may help to mitigate this behavior and thereby help to foster stronger referral networks on the continent. 

## 4. Conclusion: Building to Last

**Figure 1:**
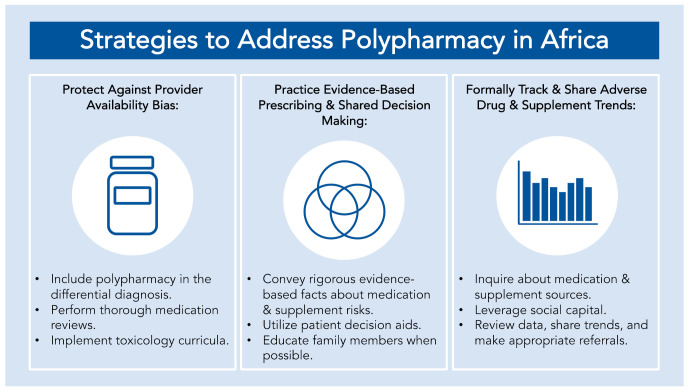
Immediately implementable strategies to address polypharmacy in Africa: by bolstering training to safeguard against provider availability biases, practicing evidence-based prescribing and shared decision making, and tracking and sharing local trends related to polypharmacy, healthcare providers and public health practitioners in Africa can better position themselves to meet the polypharmacy needs of their patients.

Although the COVID-19 pandemic has exacerbated the threat of polypharmacy on the African continent, the threat is one that is likely to persist and widen with time given Africa’s growing aging population [33,34,35,36]. Our recommendations (Figure 1) serve as immediately implementable local strategies that can address patients’ present polypharmacy care needs. Moreover, the strategies allow for ever-evolving, active interactions with patients and local healthcare ecosystems. This dynamism primes the strategies to be complementary to future advances in healthcare infrastructure and future efforts in regional and country-level health policy. With any interventions, but especially in the ever-changing COVID-19 climate, regular evaluations are necessary to determine intervention effectiveness and future directions. This frequent reevaluation will be a crucial activity for African healthcare providers and public health practitioners as they implement strategies [37]. Although this commentary was focused on immediately implementable strategies, lessons learned from these strategies may prove useful for longer-term efforts in public health policy and practice. Lastly, while our proposed strategies are focused on improving care in Africa, they may also be useful for other regions of the world that are facing similar difficulties.
